# Relationship of Serum Interleukin-10 and Its Genetic Variations with Ischemic Stroke in a Chinese General Population

**DOI:** 10.1371/journal.pone.0074126

**Published:** 2013-09-11

**Authors:** Gaoqiang Xie, Phyo Kyaw Myint, M. Justin S. Zaman, Ying Li, Liancheng Zhao, Ping Shi, Fuxiu Ren, Yangfeng Wu

**Affiliations:** 1 Peking University Clinical Research Institute, Beijing, People’s Republic of China; 2 School of Medicine and Dentistry, Institute of Applied Health Sciences, University of Aberdeen, Aberdeen, Scotland, United Kingdom; 3 Norwich Research Park Cardiovascular Research Group, Norwich Medical School, University of East Anglia, Norwich, United Kingdom; 4 Clinical Gerontology Unit, Department of Public Health and Primary Care, University of Cambridge, Cambridge, United Kingdom; 5 Stroke Research Group, Norfolk and Norwich University Hospital, Norwich, United Kingdom; 6 Department of Cardiology, James Paget University Hospital, Great Yarmouth, United Kingdom; 7 Cardiovascular Institute and Fuwai Hospital, Chinese Academy of Medical Sciences and Peking Union Medical College, People’s Republic of China; 8 Shijingshan Center for Disease Control and Prevention, Beijing, People’s Republic of China; 9 Department of Epidemiology and Biostatistics, Peking University School of Public Health, Beijing, People’s Republic of China; 10 The George Institute, Beijing, People’s Republic of China; University of Birmingham, United Kingdom

## Abstract

**Background and Purpose:**

Anti-inflammatory cytokine and its genetic variations may play an important role in the process of atherosclerosis. We assessed whether serum interleukin-10 (IL-10) and its genetic variations are associated with ischemic stroke in a Chinese general population.

**Methods:**

An epidemiological survey on cardiovascular diseases and their risk factors was carried in a general population in Beijing in 2005. Serum IL-10, IL-6, p-selectin, soluble intercellular adhesion molecule-1 and C-reactive protein were analyzed using ELISA kits, while three IL-10 Single Nucleotide Polymorphisms (SNP) (rs1800872, rs1554286 and rs3021094) were genotyped in 1475 participants.

**Results:**

A high serum IL-10 (top tertile) was significantly associated with ischemic stroke (multivariable adjusted odds ratio (OR) =0.50; 95%CI 0.31-0.81). Rs1800872 (AA vs. AC+CC genotype, OR=1.60; 1.06-2.39), rs1554286(TT vs. CT+CC genotype, OR=1.59; 1.06-2.39), and rs3021094 (CC/CA vs. AA genotype, OR=1.64; 1.04-2.60) were all significantly associated with ischemic stroke even after controlling for age, sex, smoking, systolic blood pressure, total cholesterol, glucose, body mass index and serum IL-10. The SNP score (a summary index of these SNPs) and IL-10 (top tertile) together significantly improved the discriminative power in predicting ischemic stroke by 3.3% (95%CI: 0.2-6.4, p=0.0398) compared to predictions based on conventional risk factors alone.

**Conclusions:**

The lower serum IL-10 concentration and its selected genetic variations were significantly associated with an increased likelihood of ischemic stroke in this cross-sectional study. Our results suggest that more prospective studies should be conducted to provide stronger evidence justifying the use of IL-10 and its SNPs as new biomarkers to identify a predisposition towards ischemic stroke.

## Introduction

Atherosclerosis is a disease process with an inflammatory component [1]. Thus, as an important anti-inflammatory cytokine, interleukin-10 (IL-10) has been proposed to protect against atherosclerosis progression [2,3]. Animal experiments have shown that the absence of IL-10 leads to an increased susceptibility to atherosclerosis in IL-10 knockout mice [2,3]. However, there is a dearth of information on the association between serum IL-10 and stroke in the general population. Although the sequence of chemical base pairs and most of the single nucleotide polymorphisms (SNP) of the IL-10 gene have been identified, their role in the regulation of IL-10 production and also their influence on atherosclerosis remains largely unknown.

The gene promoter is a region of an operon that acts as the initial binding site for RNA polymerase to be able to regulate the time and degree of expression of a gene [4]. Introns are non-coding sequences that interrupt eukaryotic exons and are removed from premature messenger RNAs by the spliceosomal machinery before translation [5]. Although the intron does not code protein, it has been shown to influence gene transcription by affecting the gene space structure and/or producing micro-RNA [6]. Compared with SNPs in exons and promoter region, SNPs in intron regions are less frequently studied in relation to ischemic stroke.

To date the studies which have examined the association between rs1800872, a prevalent minor allele (~27%) in the Chinese population situated in promoter region, and cardiovascular disease have yielded inconsistent results [7,8]. In addition, the effects of rs1554286 and rs3021094, minor alleles that are 28.4% and 47.8% prevalent in Chinese, which are situated in the intron region of IL-10 gene, on serum IL-10 levels and ischemic stroke have not been previously reported.

Thus, in the current study we selected rs1800872, rs1554286 and rs3021094 SNPs and examined the relationships between serum IL-10, selected IL-10 gene SNPs, and the risk of ischemic stroke using a cross-sectional study in a general Chinese population. We also examined whether these SNPs together with IL-10 improved the risk prediction of ischemic stroke above that of classical risk factors in this population. 

## Methods

### Study Subjects

The study sample was taken from the original cohort of rural Beijing participants in the People’s Republic of China/United States of America (PRC-USA) Collaborative Study of Cardiovascular and Cardiopulmonary Epidemiology. A detailed description of the goals, design and methods of PRC-USA study has been published elsewhere [9,10]. Briefly, the current study sample was drawn from a clustered random sample of 2313 men and women residing in 11 villages of the Shijingshan district of Beijing who participated in the Third survey of the PRC-USA study (1993-1994). Of the 2313 participants, 170 participants died, 105 were excluded because of missing baseline data on cardiovascular risk factors and history of cardiovascular disease (CVD), and the remaining 2038 participants were invited for the survey in 2005. Among 2038 eligible population, a total of 1756 (86.2%) were accessible and participated in the field survey.

The study was approved by the Ethical Committee of Fuwai Hospital according to the Declaration of Helsinki. Written informed consent was obtained from all participants.

### Outcome Ascertainment

People with ischemic stroke were identified using the PRC-USA definition of ischemic stroke. The diagnosis of ischemic stroke due to cerebral thrombosis or embolism was made by the participant’s general practitioner or treating nursing home physicians based on clinical findings and the computed tomography (CT).

### Genotyping

Among the field-surveyed 1756 participants, DNA was successfully drawn from 1652 (94.1%) and assayed for selected IL-10 gene polymorphisms. Genotyping of rs1800872 was performed by the polymerase chain reaction restriction fragment-length polymorphism (PCR-RFLP) method [11]. Rs1554286 and rs3021094 were genotyped using ligase detection reaction (LDR) methods in the same DNA samples.

### Cytokine Measurement

Serum cytokines such as interleukin-10(IL-10), interleukin-6 (IL-6), P-selectin, soluble intercellular adhesion molecule-1 (s-ICAM-1) and C-reactive protein (CRP) were measured using ELISA kits according to the manufacturer’s instructions (SUNBIO, China) in 1475 participants. The correlation coefficient for 18 test–retest samples was 0.96 for IL-10, 0.82 for IL-6, 0.94 for P-selectin and 0.94 for s-ICAM-1 depicting strong agreement for all (p for all <0.001). The correlation coefficient for 44 test–retest samples was 0.994 for CRP.

### Cardiovascular disease risk factor measurements

All major conventional (classical) cardiovascular risk factors were measured using the same standard protocols as in the previous PRC-USA surveys [9,10]. Current smoking was defined as having smoked at least one cigarette per day for at least the past one year. An ex-smoker was defined as an individual who previously smoked tobacco but had stopped smoking for at least one month. Hypertension was defined as systolic blood pressure (SBP) ≥140mmHg or diastolic blood pressure (DBP) ≥90 mmHg or use of antihypertensive drugs in the past 2 weeks. Diabetes was defined as a fasting glucose ≥126mg/dL or participant use of insulin or hypoglycemic agent [12]. Body mass index (BMI) was calculated as weight in kg/height in m^2^.

### Statistical analysis

The Hardy-Weinberg equilibrium was assessed using the Chi-square test (χ^2^) and the SHEsis program (http://analysis2.bio-x.cn/SHEsisMain.htm) [13]. Chi-square test (χ^2^) and logistic regression models were used for dichotomous variables. General linear models (GLM) were used to assess the effects of SNPs and other factors on serum IL-10 using SPSS for Windows version 16.0 (SPSS Inc., Chicago, IL). Multiple logistic regression models were used to assess the effects of SNPs, serum IL-10 and other factors on risk of ischemic stroke. Haplotype analyses were performed using SHEsis program [13,14].

We used both a mixed inheritance model (wild, mixed and mutant genotype as three categories in SNP variable) and a dominant inheritance model (wild/no wild genotypes as two categories in SNP variable) to assess the effects of SNPs on serum IL-10 and ischemic stroke respectively. In order to assess the combined effects of all SNPs, a combined index-SNP score was developed according to the odds ratios (ORs) of ischemic stroke observed with each SNP. A score of SNPs was calculated by summing the scores of rs1800872, rs1554286 and rs3021094 as described in **Supplementary A** in File S1. Among the 1475 participants who had cytokine measurement, there were 412, 440, 144 and 479 persons whose SNP score was 0, 1, 2 and 3 respectively. We combined those with score 1 and 2 as one group in order to increase statistical power. In the models, two dummy variables were used to assess the SNP score: score 1 or 2 (yes/no) and score 3 (yes/no).

To analyze the additive contribution of IL-10 and its SNPs in predicting the risk of ischemic stroke, we created three logistic models using the data of 1475 participants (**Supplementary B** in File S1)—the first model used IL-10(yes/no top tertile) and its SNPs score only, the second model used conventional risk factors only and the third model used conventional risk factors plus IL-10(yes/no top tertile) and its SNP score. Then, the predicted absolute risk (AR) or probability (P) of ischemic stroke for each participant was calculated using these logistic regression coefficients from these models in the following equations:

Model 1: AR_1_ = Odds_1_/ (Odds_1_+1), where Odds_1_ = EXP {(-0.65×[IL10_top_tertile(yes/no)] +0.20×[SNPscore_1_2(yes/no)] + 0.69×[SNPscore_3(yes/no)]-2.71};

Model 2: AR_2_ = Odds_2_/ (Odds_2_+1), where Odd_2_ = EXP {0.05×[age(yr)]-0.44×[sex(man/woman)] -0.07×[smoking(yes/no)] +0.56×[ex-smoking(yes/no)] +0.01×[SBP (mmHg)] +0.003×[TC(mg/dl)]-0.002×[glucose(mg/dl)]-0.004×[BMI(kg/m^2^)]-6.37};

Model 3: AR_3_ = Odd_3_/ (Odd_3_+1), where Odd_3_ = EXP {0.05×[age(yr)]-0.48×[sex(man/woman)] -0.73×[IL10_top_tertile(yes/no)] +0.17×[SNPscore_1_2(yes/no)] +0.77×[SNPscore_3(yes/no)] -0.06×[smoking(yes/no)] +0.57×[ex-smoking(yes/no)] +0.01×[SBP (mmHg)] +0.004×[TC(mg/dl)]-0.001×[glucose(mg/dl)]-0.005×[BMI(kg/m^2^)]-6.84}.

In all models, yes was 1 and no was 0, EXP was a function that EXP(numexpr)=e^numexpr^, where e≈2.71828.

Receiver operating characteristic curve (ROC) analysis was used to compare the usefulness of the models in predicting the absolute risk of ischemic stroke. Equality of the area under the curves (AUC, or c-statistics) was tested using the ROCCONTRAST function in logistics procedure in SAS 9.2. A two-sided p value of <0.05 was regarded as statistically significantly different.

## Results

### Characteristics of the participants with and without ischemic stroke

Of the 1475 participants with complete data on SNPs, other biomarkers and risk factors, 106 (7.2%) had had an ischemic stroke. Compared to those without ischemic stroke, those who had had an ischemic stroke were older, more likely to be men, had higher systolic/diastolic blood pressure, higher triglyceride levels, lower high density lipoprotein levels, a more frequent history of hypertension and myocardial infarction, were more likely to be on anti-inflammatory drugs and lipid lowering drugs and had lower frequencies of the CC genotype of rs1800872 and AA genotype of rs3021094 (all p<0.05) (Table **1**). The prevalence of ischemic stroke increased with older age in both men and women (**Supplementary C **in File S1).

**Table 1:** Sample characteristics and comparison between those participants with and without ischemic stroke.

Variables	Cases (N=106)			Controls(N=1369)		p values*
	Mean	SD		Mean	SD	
Age (years)	63.3	7.8		59.4	8.0	<0.001
Body mass index(kg/m^2^)	26.2	3.0		27.2	18.8	0.561
Systolic blood pressure (mmHg)	144.8	19.5		138.1	20.6	0.001
Diastolic blood pressure (mmHg)	84.5	10.3		82.3	10.6	0.036
Total cholesterol (mg/dl)	204.4	36.7		200.6	35.9	0.295
Triglyceride	162.2	102.8		139.8	88.8	0.013
high density lipoprotein cholesterol	46.0	10.4		49.2	12.3	0.010
Glucose (mg/dl)	104.0	33.6		104.9	38.3	0.829
C-reactive protein (ug/ml)	3.79	3.32		3.47	3.78	0.395
P-selectin(ng/ml)	0.19	0.21		0.16	0.18	0.061
s-ICAM-1(ng/ml)^	655.1	222.6		669.1	259.7	0.692
Interleukin-6(pg/ml)	18.1	24.7		17.9	20.9	0.922
Interleukin-10(pg/ml)	33.1	28.6		34.5	39.1	0.725
						
	Cases	%		Controls	%	p values#
Interleukin-10						
1^st^ tertile§	36	34.0		452	33.0	
2^nd^ tertile	47	44.3		448	32.7	
3^rd^ tertile	23	21.7		469	34.3	0.013
Men	49	46.2		466	34.0	0.011
Current smoking	29	27.4		407	29.7	0.606
Current drinking	24	22.6		327	23.9	0.772
Diabetes	23	21.7		201	14.7	0.053
Hypertension	87	82.1		688	50.3	<0.001
Myocardial infarction	10	9.4		41	3.0	<0.001
On anti-inflammatory drugs	44	41.5		226	16.5	<0.001
On lipid lowering drugs	7	6.6		41	3.0	0.044
CC genotype of rs1800872	52	49.1		808	59.0	0.045
TT genotype of rs1554286	52	49.1		545	39.8	0.062
AA genotype of rs3021094	28	26.4		494	36.1	0.045

* Using t-test.

#Using χ^2^-test.

^ s-ICAM-1: soluble intercellular adhesion molecule-1.

§ Participants were divided into 3 equal tertiles of IL-10 by 10-year age groups.

### Distribution and Linkage Correlations of SNPs

The distributions of three IL-10 gene SNPs were consistent with the Hardy-Weinberg equilibrium in those 1369 without ischemic stroke (p=0.28 for rs1800872, p=0.10 for rs1554286, and p=0.25 for rs3021094) and in those 106 with ischemic stroke (p=0.39 for rs1800872, p=0.97 for rs1554286, and p=0.10 for rs3021094). There were significant linkage correlations among rs1800872, rs1554286 and rs3021094 (r^2^=0.76 for rs1800872 vs. rs1554286, 0.30 for rs1800872 vs. rs3021094, and 0.40 for rs1554286 vs. rs3021094, all p<0.001) using SHEsis software [13,14]. The C allele of rs1800872 was significantly linked with C allele of rs1554286 and A allele of rs3021094 (p<0.001).

### Serum IL-10 and ischemic stroke

The mean level of serum IL-10 in the total sample was 34.4±38.5pg/ml (34.0±23.8 pg/ml in men, and 34.6±44.4pg/ml in women), which was significantly positively correlated with age(Spearman R=0.15, p<0.001). Uni-variable analyses showed that the mean IL-10 was not significantly different between patients with ischemic stroke and controls (Table **1**). In order to adjust for the confounding effects of age and sex, the sample was divided into three equal tertiles of IL-10 by 10-year age groups. The sample was stratified into 40-, 50-, 60-, and 70-year age groups in both men and women. Then each group was divided into three equal tertiles of IL-10. We then combined all first tertiles of all age-sex-groups as first tertile. The same approach was used to categorise participants into second and third tertiles. The distribution of age-sex-adjusted tertiles of IL-10 was significantly different between patients with ischemic stroke and controls (p=0.013) (Table **1**). Compared to those of the 1^st^+2^nd^ tertile of IL-10, the odds ratio (OR) for ischemic stroke was significantly decreased in those of the 3^rd^ tertile of IL-10 (OR=0.50, 95%CI: 0.31-0.81, p=0.005) after adjusting for age, sex, smoking, systolic blood pressure, total cholesterol, glucose and body mass index (Table **2**).

**Table 2:** Relationship between interleukin-10 (IL-10) and ischemic stroke in 1475 participants.

IL-10(pg/ml)	Sample site	Cases	Rates (%)	Odds ratios(95%CI)*
Per 1 SD#(38) increase	1475	106	7.2%	0.89 (0.66-1.19)
Tertile of IL-10§				
1^st^ tertile	488	36	7.4	1
2^nd^ tertile	495	47	9.5	1.27 (0.80-2.01)
3^rd^ tertile	492	23	4.7	0.57 (0.33-0.99)
p for trends*			0.013	0.011
1^st^ +2^nd^ tertile	983	83	8.4	1
3^rd^ tertile	492	23	4.7	0.50 (0.31-0.81)
p values*			0.008	0.005

* Odds ratios (95%CI) and p values were calculated by multiple logistic models after adjusting for age, sex, smoking, systolic blood pressure, cholesterol, glucose, and body mass index.

# SD was standard deviation of IL-10.

§ Participants were divided into 3 equal tertiles of IL-10 by 10-year age groups.

### IL-10 SNPs and ischemic stroke

The odds ratio of ischemic stroke was significantly increased in those with AA versus AC+CC genotype of rs1800872 (OR = 1.54,95%CI:1.03-2.30,p=0.036), TT versus CC+CT genotype of rs1554286 (OR=1.54, 95%CI: 1.02-2.30, p=0.038), and CC/CA versus AA genotypes of rs3021094 (OR = 1.62,95%CI: 1.03-2.54, p=0.038) after adjustments were made for age, sex, smoking, systolic blood pressure, cholesterol, glucose, and body mass index(Table **3**). The addition of serum IL-10(yes/no top tertile categorical variable) into the model remained these ORs significant (Table **3**). Haplotype analyses found that ATA and ATC haplotypes of rs1800872, rs1554286, and rs3021094 were significantly associated with ischemic stroke: a decreased odds ratio with ATA (OR=0.68, 95%CI: 0.46-0.99, p=0.04) and an increased odds ratio with ATC (OR=1.59, 95%CI: 1.20-2.11, p=0.001) (Table **4**).

**Table 3:** IL-10 SNPs and risk of ischemic stroke in 1475 participants.

**SNPs**	N	cases	**%**	Odds Ratios (95%CI)*	Odds Ratios (95%CI)#
**rs1800872**					
Mixed Inheritance Model					
AA	615	54	8.8%	1.39 (0.72-2.68)	1.42 (0.73-2.76)
AC	682	40	5.9%	0.88 (0.44-1.73)	0.86 (0.44-1.71)
CC	178	12	6.7%	1	1
P for trends			0.124†	0.104	0.072
Dominant Inheritance Model					
AA	615	54	8.8%	1.54 (1.03-2.30)	1.60 (1.06-2.39)
CC+AC	860	52	6.0%	1	1
P values			0.045‡	0.036	0.024
**rs1554286**					
Mixed Inheritance Model					
TT	597	52	8.7%	1.29 (0.70-2.37)	1.33 (0.72-2.46)
CT	676	39	5.8%	0.79 (0.42-1.48)	0.79 (0.42-1.48)
CC	202	15	7.4%	1	1
P for trends			0.127†	0.092	
Dominant Inheritance Model					
TT	597	52	8.7%	1.54 (1.02-2.30)	1.59 (1.06-2.39)
CC+CT	878	54	6.2%	1	1
P values			0.062‡	0.038	0.025
**rs3021094**					
Mixed Inheritance Model					
AA	522	28	5.4%	1	1
AC	679	47	6.9%	1.31 (0.80-2.14)	1.33 (0.81-2.17)
**CC**	274	31	11.3%	2.48 (1.44-4.29)	2.59 (1.49-4.48)
P for trends			0.008†	0.003	0.002
Dominant Inheritance Model					
AA	522	28	5.4%	1	1
CC+AC	953	78	8.2%	1.62 (1.03-2.54)	1.64 (1.04-2.60)
P values			0.045‡	0.038	0.056
**Scores of SNPs^**					
0	412	22	5.3%	1	1
1-2	584	37	6.3%	1.17 (0.67-2.03)	1.19 (0.68-2.07)
3	479	47	9.8%	2.08 (1.22-3.55)	2.16 (1.26-3.70)
P for trends			0.021†	0.008	0.006

* Odds ratios (95%CI) and p values were calculated by multiple logistic models after adjusting for age, sex, smoking, systolic blood pressure, cholesterol, glucose, and body mass index.

# Odds ratios(95%CI) and p values were calculated by multiple logistic models after adjusting for age, sex, smoking, systolic blood pressure, cholesterol, glucose, body mass index, and IL-10(yes/no top tertile).

† P for trends were calculated by Cochran-Armitage trend test.

‡ P values were calculated by chi-square tests.

^ Score of SNPs was summary of scores of rs1800872 (if AA, then score=1, if CC+AC, then score=0), rs1554286 (if TT, then score=1, if CC+CT, then score=0), and rs3021094 (if CC+AC, then score=1, if AA, then score=0). Those with score value 1 and 2 were combined into one group in order to increase the statistic power.

**Table 4:** Haplotype analysis for loci of rs1800872, rs1554286, rs3021094 and risk of ischemic stroke.

Haplotypes (rs1800872/rs1554286/rs3021094)	Case (%)	Control (%)	OR(95%CI)#	P for OR#
A C A	5 (0.023)	100 (0.037)	0.62 (0.25-1.55)	0.302
A C C*	1 (0.005)	1 (0.000)	-	
A T A	34 (0.159)	596 (0.217)	0.68 (0.46-0.99)	0.040
A T C	108 (0.504)	1067 (0.389)	1.59 (1.20-2.11)	0.001
C C A	63 (0.294)	910 (0.332)	0.83 (0.61-1.12)	0.234
C T A*	1 (0.005)	14 (0.005)	-	
C T C*	0 (0.000)	50 (0.018)	-	

# Odds ratios (95%CI) and p values were calculated by SHEsis software online (http://analysis2.bio-x.cn/myAnalysis.php) [13,14].

* All those frequency<0.03 will be ignored in analysis.

### Effects of IL-10 gene polymorphisms on serum IL-10

**Figure 1:**
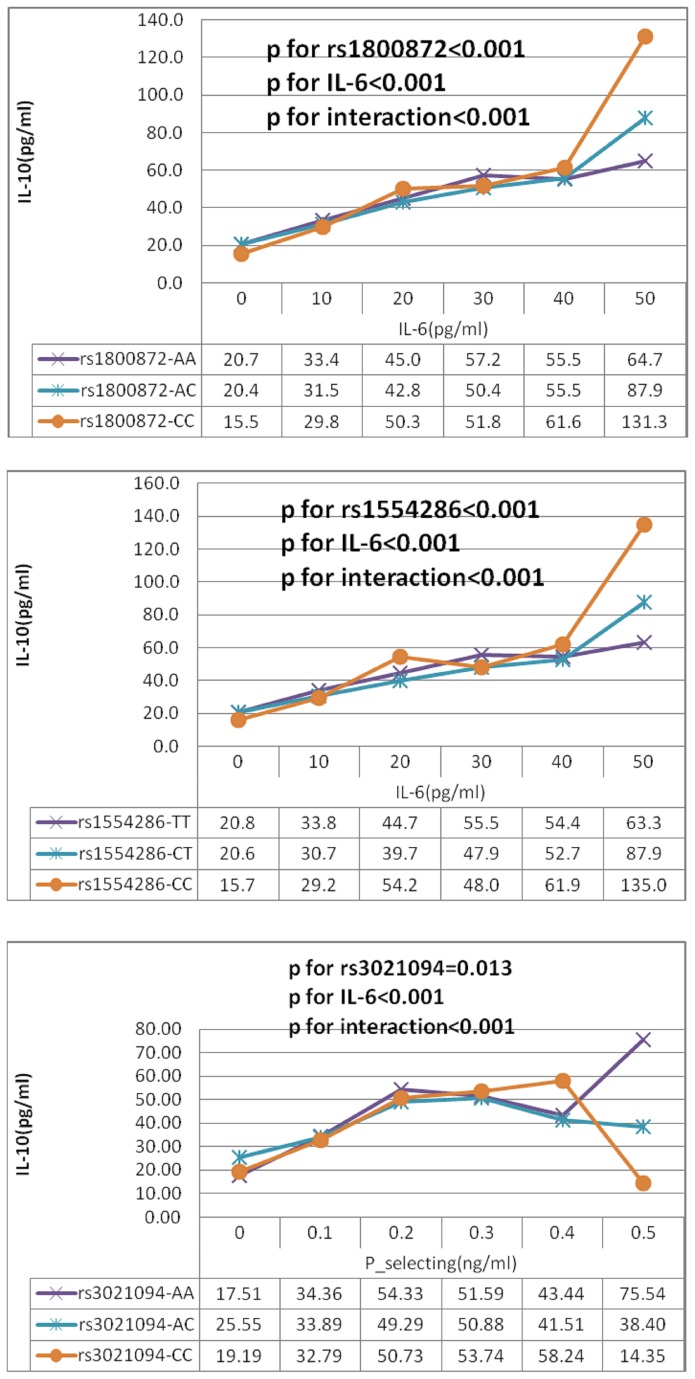
Age-adjusted IL-10 was significantly influenced by interaction genotypes (rs1800872, rs1554286, and rs3021094) with IL-6 and P-selectin in 1475 participants. Age-adjusted IL-10 and P values were calculated by multiple general linear models (GLM) after adjusting for age.

In both univariable and age and sex adjusted general linear models serum IL-10 levels were not significantly associated with any polymorphisms (**Supplementary D **in File S1). However, serum IL-10 level was significantly influenced by interactions: rs1800872×age, rs1800872×IL-6, rs1554286×IL-6, rs1554286×P-selectin, rs3021094× P-selectin (p for all <0.05) in multiple general linear models (**Supplementary E **in File S1). Figure **1** shows that IL-10 level increased with IL-6 more steeply in those with the CC genotype of rs1800872, CC genotype of rs1554286 and with P-selectin in those with the AA genotype of rs3021094. Thus, when these interactions were added into the models, serum IL-10 was significantly associated with rs1800872, rs1554286 and rs3021094 (**Supplementary D **in File S1).

### The value of adding IL-10 and its SNPs to classical risk model for ischemic stroke

**Figure 2:**
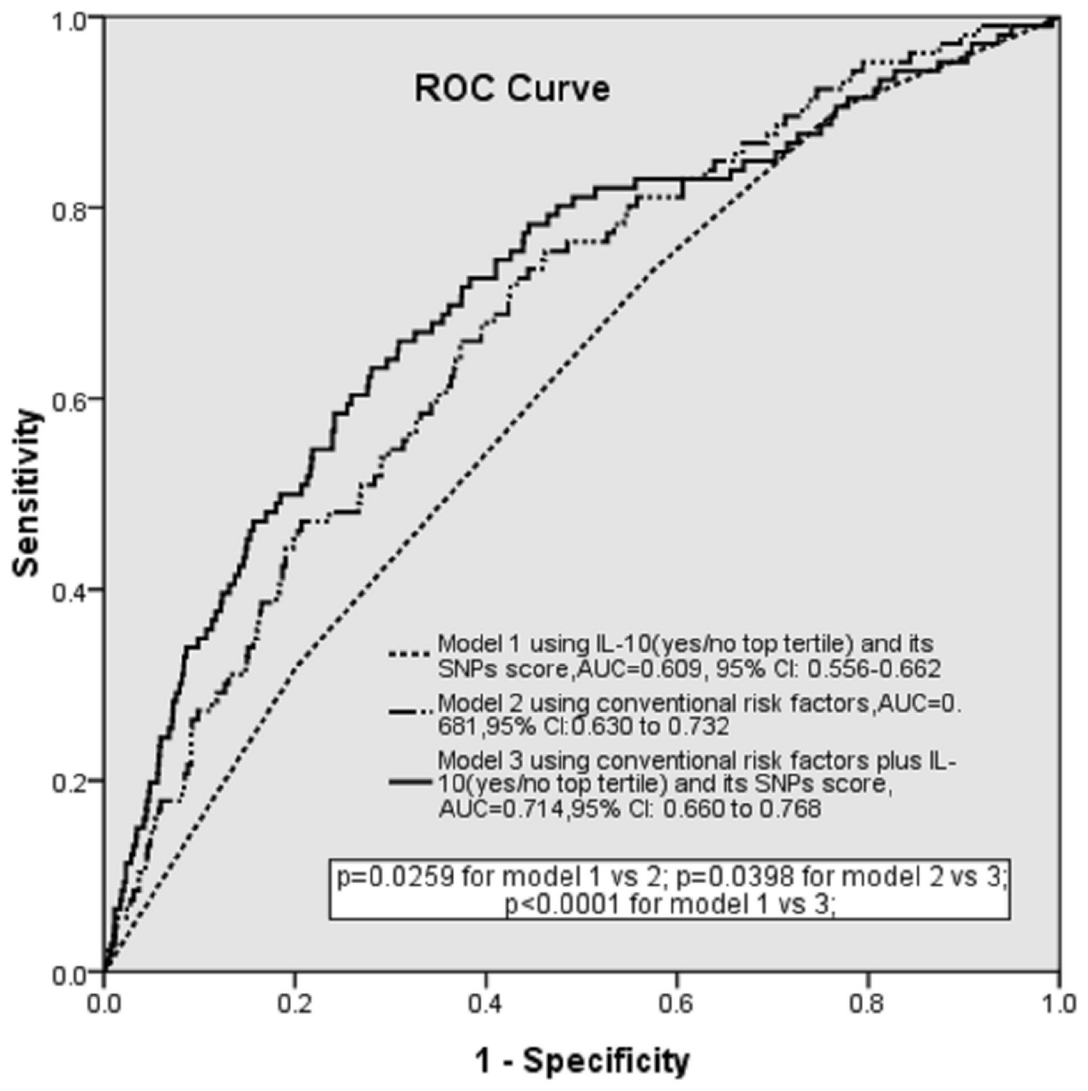
Receiver operating characteristic (ROC) curves and corresponding areas under the curve (AUCs) comparing the discriminative power of IL-10(yes/no top tertile) and its SNP score only, the conventional risk score alone and their combination.

Model 1 containing IL-10 (yes/no top tertile) and SNPs scores significantly predicted the likelihood of ischemic stroke but with a modest AUC of 0.609 (95% CI 0.556-0.662, p<0.001) (Figure 2). However, the addition of IL-10 (yes/no top tertile) and SNPs score in model 3 resulted in a significant increase in AUC from 0.681 (95%CI 0.630 to 0.732) using model 2 (conventional risk factors only) to 0.714 (95% CI 0.660 to 0.768). The absolute increase in AUC was 3.3% (95%CI 0.2 to 6.4, p=0.0398).

## Discussion

Our results show that a higher serum IL-10 level may be associated with a reduced risk of ischemic stroke in a general population. This work provides new evidence backing up the hypothesis of the protective effects of IL-10 on the atherosclerotic process. Ross first proposed that atherosclerosis is an inflammatory disease and not merely a process of the accumulation of lipids within the artery wall in 1999 [1]. Since then, many studies have been conducted to investigate the associations between markers of inflammation and ischemic cardiovascular diseases. Interleukin(IL)-10, as an important anti-inflammatory T-cell cytokines, had been identified as a cytokine that regulates at least three different types of macrophages which play an important role in atherosclerosis [1]. Animal experiments have shown the protective effects of IL-10 on atherosclerotic lesion formation in IL-10 transgenic mice model and IL-10 knockout mice model [2,3]. Moreover, in patients with acute coronary syndromes it has been demonstrated that elevated serum IL-10 levels are associated with a significantly improved outcome [15]. In those > =85 years, lower IL-10 production in lipopolysaccharide-stimulated whole-blood samples have been shown to be significantly associated with increased incident fatal stroke [16]. Compared with stimulated IL-10 production, serum IL-10 is easier to assess and thus may be incorporated in clinical practice to risk stratify the likelihood of ischemic event.

Although the sequence of chemical base pairs and most SNPs of the IL-10 gene have been identified [17], their role in the aetiology of ischemic disease in humans is still largely unknown. Rs1800872 is one of the most studied SNP in the promoter region of IL-10 gene [7]. A large case-control (1107 cases, 1082 controls) study did not show a significant association between rs1800872 and the risk of myocardial infarction [7], but other studies have shown that the haplotype associated with the -592A allele of rs1800872 is associated with a higher risk of ischemic cardiovascular events [8], and with postoperative cardiovascular events in patients with peripheral artery disease receiving elective surgical revascularization [18] and with coronary artery lesions in the acute stage of Kawasaki disease [19]. Our study confirms that the A allele of rs1800872 is significantly associated with higher risk of ischemic stroke in a general population.

The potential mechanism of this observed association may be that rs1800872 influences the regulation of the production of IL-10. An extended twin study found that 50-70% of the variance in production level of IL-10 is genetically determined [20]. A recent small-sample study showed that serum IL-10 was significantly higher in ten rheumatoid arthritis patients carrying the C allele of rs1800872(-592A/C) [21]. We show that serum IL-10 level increases with serum IL-6 more rapidly in those with AA genotype of rs1800872 (p for interaction<0.001) (Figure 1). Thus, although it is not known whether the rs1800872 is alone in this association or whether it is involved in a linkage disequilibrium with another variant, our and previous studies suggest that it is an important genetic marker that predisposes to ischemic stroke.

Although an intron does not code protein, it has been shown to influence gene transcription by affecting gene space structure and/or produce micro-RNA [6]. However, compared with SNPs in exons and promoter region, SNPs in the intron region are less frequently studied in relation to ischemic stroke [7]. We show here that rs1554286 and rs3021094, located in the intron region and highly prevalent in Chinese populations, were significantly associated with ischemic stroke. To our knowledge this association has not been previously reported. We suggest here that they are important genetic markers that predispose to ischemic stroke and we propose a need for more work to explore their potential role in the aetiology of stroke.

In order to assess the combined effects of these SNPs (rs1800872, rs1554286 and rs3021094), we developed a combined index-an SNP score derived from the ORs of each SNP - and found that this SNP score was significantly associated with ischemic stroke. We also conducted haplotype analyses and confirmed the combined effects of these three SNPs, showing that the haplotype ATC with -592A allele of rs1800872 was significantly associated with ischemic stroke as previously reported [8]. We found that the influences of serum IL-10 and the above three SNPs on ischemic stroke were independent from each other. Therefore, we further assessed the additional value of serum IL-10 and SNPs in predicting ischemic stroke over and above that of a prognostic model with conventional risk factors alone. We have shown that the addition of serum IL-10 and the SNPs score provides a significantly better prediction of ischemic stroke than conventional risk factors alone. The absolute increase in AUC was 3.3%, comparable to that of systolic blood pressure (data were not shown) and also comparable to half of the effect size of total burden score of carotid atherosclerosis which is predictive of ischemic cardiovascular events [22]. Thus, IL-10 and its three SNPs have a potentially important role in clinical practice for the estimation of an individual’s predisposition to ischemic stroke.

This study has some limitations. Firstly, we excluded 281 participants (16% of the potentially eligible total sample of 1756) due to a lack of volume of DNA or serum resulting in no genotyping being possible or no serum cytokine measurement being possible. Excluded subjects had a higher risk of stroke (they were older and had higher SBPs) (**Supplementary F **in File S1). Exclusion of high risk participants however is only likely to attenuate the strength of the associations observed because the association between IL-10 and SNPs and ischemic stroke is stronger in high-risk participants than in low-risk participants in the present study (data were not shown). Secondly, there were only 106 participants who had stroke- thus there is the potential to have underestimated the associations we found. Finally, the models and results may be only applicable for this Chinese population and would need to be assessed separately in other populations.

Despite these limitations, our study provides new evidence that lower serum IL-10 and its genetic variations (AA genotype of rs1800872, TT genotype of rs1554286, and CA/CC genotype of rs3021094) are associated with an increased likelihood of ischemic stroke. Our results suggest that more prospective studies should be conducted to provide more data to justify the use of IL-10 and its SNPs as new biomarkers to identify an increased predisposition to ischemic stroke.

## Supporting Information

File S1 Supplementary A in file S1. Scores of SNPs (rs1800872, rs1554286, and rs3021094). Supplementary B in file S1. Models 1, 2, 3. Supplementary C in file S1. Prevalence of ischemic stroke in men and women by different age group. Supplementary D in file S1. IL-10 SNPs and serum IL-10 in 1475 participants. Supplementary E in file S1. Effects of interactions between genotypes and factors on serum IL-10 level. Supplementary F in file S1. Sample characteristics and comparison between those included and those excluded.
(DOC)
