# Supplementary material for: Relationship of Serum Interleukin-10 and Its Genetic Variations with Ischemic Stroke in a Chinese General Population
Source: PLoS One. 2013 Sep 11;8(9):e74126. doi: 10.1371/journal.pone.0074126 (PMC3770660; doi:10.1371/journal.pone.0074126)
Supplement: File S1 — Supplementary A in file S1. Scores of SNPs (rs1800872, rs1554286, and rs3021094). Supplementary B in file S1. Models 1, 2, 3. Supplementary C in file S1. Prevalence of ischemic stroke in men and women by different age group. Supplementary D in file S1. IL-10 SNPs and serum IL-10 in 1475 participants. Supplementary E in file S1. Effects of interactions between genotypes and factors on serum IL-10 level. Supplementary F in file S1. Sample characteristics and comparison between those included and those excluded. (DOC) [file pone.0074126.s001.doc]

Supplementary A. Scores of SNPs (rs1800872, rs1554286, and rs3021094)

| SNPs | Scores |
| --- | --- |
| rs1800872 |  |
| AA | 1 |
| CC or AC | 0 |
| rs1554286 |  |
| TT | 1 |
| CC+CT | 0 |
| rs3021094 |  |
| CC or AC | 1 |
| AA | 0 |

**Supplementary B. Model 1,2 and 3**

**Models 1,2, and 3 are shown as following.**

Model 1: ln(Odds)=$\ln\left(\frac{p}{1-p}\right)$=-0.65×([IL10_top_tertile(yes/no)] +0.20×[SNPscore_1_2(yes/no)]+ 0.69×[SNPscore_3(yes/no)]-2.71;

Model 2: ln(Odds)=$\ln\left(\frac{p}{1-p}\right)$= 0.05×[age(yr)]-0.44×[sex(man/woman)] -0.07×[smoking(yes/no)] +0.56×[ex-smoking(yes/no)] +0.01×[SBP (mmHg)] +0.003×[TC(mg/dl)]-0.002×[glucose(mg/dl)]-0.004×[BMI(kg/m^2^)]-6.37;

Model 3: ln(Odds)=$\ln\left(\frac{p}{1-p}\right)$=0.05×[age(yr)]-0.48×[sex(man/woman)] -0.73×[IL10_top_tertile(yes/no)] +0.17×[SNPscore_1_2(yes/no)]+0.77×[SNPscore_3(yes/no)] -0.06×[smoking(yes/no)]+0.57×[ex-smoking(yes/no)]+0.01×[SBP (mmHg)] +0.004×[TC(mg/dl) ]-0.001×[glucose(mg/dl)]-0.005×[BMI(kg/m^2^)]-6.84.

Where *p* is probability of ischemic stroke

Supplementary C. Prevalence of ischemic stroke in men and women by different age group

| Ages(years) | Men | | |  | Women | | |
| --- | --- | --- | --- | --- | --- | --- | --- |
|  | Total | Cases of ischemic stroke | Rates of ischemic stroke(%) |  | Total | Cases of ischemic stroke | Rates of ischemic stroke(%) |
| 40-49 | 93 | 3 | 3.2% |  | 130 | 3 | 2.3% |
| 50-59 | 187 | 18 | 9.6% |  | 314 | 11 | 3.5% |
| 60-69 | 147 | 20 | 13.6% |  | 422 | 29 | 6.9% |
| 70-79 | 88 | 8 | 9.1% |  | 94 | 14 | 14.9% |
| Total | 515 | 49 | 9.5% |  | 960 | 57 | 5.9% |
| P for trends |  |  | 0.021 |  |  |  | <0.001 |

Supplementary D. IL-10 SNPs and serum IL-10 in 1475 participants

| **SNP** | N | Age-sex-adjusted Mean of serum IL-10 concentration (95%CI) | **p values*** | **p values** |
| --- | --- | --- | --- | --- |
| rs1800872 |  |  |  |  |
| Mixed Inheritance Model |  |  |  |  |
| AA | 615 | 35.1 (32.0-38.2) |  |  |
| AC | 682 | 33.5 (30.5-36.5) |  |  |
| CC | 178 | 34.9 (29.3-40.6) | 0.742 | <0.001# |
| Dominant Inheritance Model |  |  |  |  |
| AA | 615 | 35.1 (32.0-38.2) |  |  |
| CC+AC | 860 | 33.8 (31.2-36.4) | 0.524 | 0.002# |
| **rs1554286** |  |  |  |  |
| Mixed Inheritance Model |  |  |  |  |
| TT | 597 | 35.0(31.8-38.1) |  |  |
| CT | 676 | 33.7(30.7-36.7) |  |  |
| CC | 202 | 34.5 (29.2-39.9) | 0.836 | <0.001※ |
| Dominant Inheritance Model |  |  |  |  |
| TT | 597 | 35.0 (31.8-38.1) |  |  |
| CC+CT | 878 | 33.9 (31.3-36.5) | 0.595 | 0.001※ |
| **Rs3021094** |  |  |  |  |
| Mixed Inheritance Model |  |  |  |  |
| AA | 522 | 33.1 (29.8-36.5) |  |  |
| AC | 679 | 35.2 (32.2-38.1) |  |  |
| **CC** | 274 | 34.5 (29.9-39.1) | 0.657 | <0.001^ |
| Dominant Inheritance Model |  |  |  |  |
| AA | 522 | 33.1(29.8-36.5) |  |  |
| CC+AC | 953 | 35.0(32.5-37.5) | 0.375 | <0.001^ |

*Means and p values were calculated by general linear models (GLM) after adjusting for age and sex;

#Means and p values were calculated by general linear models (GLM) after adjusting for age, sex, smoking, systolic blood pressure , total cholesterol, glucose, body mass index, IL-6, and interaction between IL-6 and rs1800872.

※Means and p values were calculated by general linear models (GLM) after adjusting for age, sex, smoking, systolic blood pressure , total cholesterol, glucose, body mass index, IL-6, and interaction between IL-6 and rs1554286.

^ Means and p values were calculated by general linear models (GLM) after adjusting for age, sex, smoking, systolic blood pressure , total cholesterol, glucose, body mass index, P-selectin, and interaction between P-selectin and rs3021094.

Supplementary E. Effects of interactions between genotypes and factors on serum IL-10 level

| Interaction of genotypes with: | rs1800872  (AA vs. AC+CC) | |  | rs1554286  (TT vs. CT+CC) | |  | rs3021094  (AA vs. AC+CC) | |
| --- | --- | --- | --- | --- | --- | --- | --- | --- |
|  | B | p value for interaction* |  | B | p value for interaction* |  | B | p value for interaction* |
| Men | -3.88 | 0.343 |  | -4.30 | 0.297 |  | -0.91 | 0.828 |
| Age (years) | -0.51 | 0.035 |  | -0.46 | 0.060 |  | -0.13 | 0.607 |
| Total cholesterol (mg/dl) | 0.01 | 0.117 |  | 0.03 | 0.648 |  | 0.01 | 0.844 |
| CRP(ug/ml) | -0.20 | 0.702 |  | -0.19 | 0.710 |  | -0.24 | 0.783 |
| Interleukin-6(pg/ml) | 0.36 | <0.001 |  | 0.38 | <0.001 |  | 0.04 | 0.651 |
| P-selectin (ng/ml) | 12.8 | 0.257 |  | 26.2 | 0.029 |  | -86.6 | <0.001 |

*p values for interaction were calculated by general linear models (GLM) after adjusting for age, sex, smoking, systolic blood pressure , total cholesterol, glucose, body mass index, CRP, interleukin-6, and P-selectin.

Supplementary F: Sample characteristics and comparison between those included and those excluded

| Variables | Study  Subjects (N=1475) | |  | Excluded subjects | | | p values* |
| --- | --- | --- | --- | --- | --- | --- | --- |
|  | Mean | SD |  | N | Mean | SD |  |
| Age (years) | 59.7 | 8.1 |  | 278 | 62.6 | 8.4 | <0.001 |
| Body mass index | 27.2 | 18.1 |  | 277 | 25.9 | 4.0 | 0.253 |
| Systolic blood pressure (mmHg) | 138.6 | 20.6 |  | 278 | 144.5 | 22.6 | <0.001 |
| Diastolic blood pressure (mmHg) | 82.4 | 10.6 |  | 278 | 82.9 | 9.9 | 0.530 |
| Total cholesterol (mg/dl) | 200.9 | 35.9 |  | 278 | 197.3 | 36.9 | 0.126 |
| TG | 141.4 | 90.0 |  | 278 | 142.1 | 83.9 | 0.904 |
| HDL-c | 49.0 | 12.2 |  | 278 | 47.8 | 11.2 | 0.119 |
| Glucose (mg/dl) | 104.8 | 38.0 |  | 278 | 108.9 | 47.0 | 0.175 |
| CRP(ug/ml) | 3.5 | 3.7 |  | 277 | 4.5 | 4.5 | 0.001 |
| Interleukin-6(pg/ml) | 17.9 | 21.2 |  | 263 | 21.3 | 20.1 | 0.016 |
| Interleukin-10(pg/ml) | 34.4 | 38.5 |  | 277 | 38.6 | 44.7 | 0.148 |
| P-selectin(ng/ml) | 0.16 | 0.18 |  | 112 | 0.12 | 0.09 | 0.012 |
| s-ICAM-1(ng/ml) | 668.1 | 351.6 |  | 112 | 687.1 | 388.6 | 0.586 |
|  |  |  |  |  |  |  |  |
|  | Cases | % |  | N | Cases | % | p values# |
| Men | 515 | 34.9 |  | 280 | 106 | 37.9 | 0.304 |
| Current smoking | 436 | 29.6 |  | 280 | 90 | 32.1 | 0.387 |
| Current drinking | 351 | 23.8 |  | 280 | 67 | 23.9 | 0.962 |
| Diabetes | 224 | 15.2 |  | 280 | 50 | 17.9 | 0.259 |
| Hypertension | 775 | 52.5 |  | 280 | 189 | 67.5 | <0.001 |
| Myocardial infarction | 51 | 3.5 |  | 280 | 11 | 3.9 | 0.696 |
| Ischemic stroke | 106 | 7.2 |  | 280 | 21 | 7.5 | 0.853 |
| On anti-inflammatory drugs | 270 | 18.3 |  | 280 | 45 | 16.1 | 0.372 |
| On lipid lowering drugs | 48 | 3.3 |  | 280 | 11 | 3.9 | 0.566 |
| rs1800872CC | 615 | 41.7 |  | 177 | 83 | 46.9 | 0.186 |
| rs1554286TT | 597 | 40.5 |  | 177 | 78 | 44.1 | 0.358 |
| rs3021094AA | 522 | 35.4 |  | 177 | 57 | 32.2 | 0.401 |

*Using t-test

#Using χ^2^-test
